# Impact of acute sleep deprivation on dynamic functional connectivity states

**DOI:** 10.1002/hbm.24855

**Published:** 2019-11-04

**Authors:** Changhong Li, Judith Fronczek‐Poncelet, Denise Lange, Eva Hennecke, Tina Kroll, Andreas Matusch, Daniel Aeschbach, Andreas Bauer, Eva‐Maria Elmenhorst, David Elmenhorst

**Affiliations:** ^1^ Institute of Neuroscience and Medicine (INM‐2), Forschungszentrum Jülich Jülich Germany; ^2^ Department of Sleep and Human Factors Research Institute of Aerospace Medicine, German Aerospace Center Cologne Germany; ^3^ Division of Sleep Medicine Harvard Medical School, Sleep Division Boston Massachusetts; ^4^ Department of Neurology, Medical Faculty Heinrich‐Heine‐University Düsseldorf Düsseldorf Germany; ^5^ Division of Medical Psychology Rheinische Friedrich‐Wilhelms‐University Bonn Bonn Germany

**Keywords:** acute sleep deprivation, dynamic connectivity states, light sleep/drowsiness, resting‐state fMRI, test–retest reliability

## Abstract

Sleep deprivation (SD) could amplify the temporal fluctuation of spontaneous brain activities that reflect different arousal levels using a dynamic functional connectivity (dFC) approach. Therefore, we intended to evaluate the test–retest reliability of dFC characteristics during rested wakefulness (RW), and to explore how the properties of these dynamic connectivity states were affected by extended durations of acute sleep loss (28/52 hr). We acquired resting‐state fMRI and neuropsychological datasets in two independent studies: (a) twice during RW and once after 28 hr of SD (*n* = 15) and (b) after 52 hr of SD and after 14 hr of recovery sleep (RS; *n* = 14). Sliding‐window correlations approach was applied to estimate their covariance matrices and corresponding three connectivity states were generated. The test–retest reliability of dFC properties demonstrated mean dwell time and fraction of connectivity states were reliable. After SD, the mean dwell time of a specific state, featured by strong subcortical–cortical anticorrelations, was significantly increased. Conversely, another globally hypoconnected state was significantly decreased. Subjective sleepiness and objective performances were separately positive and negative correlated with the increased and decreased state. Two brain connectivity states and their alterations might be sufficiently sensitive to reflect changes in the dynamics of brain mental activities after sleep loss.

## 1 INTRODUCTION

Many prior studies (Goel, Rao, Durmer, & Dinges, 2009; Harrison & Horne, 2000; Louca & Short, 2014; Pilcher & Huffcutt, 1996) demonstrated that acute sleep deprivation (SD), even a single night of sleep loss, could induce substantial impairments of cognitive performance, memory abilities, and emotion regulations. Meanwhile, a growing body of research (Chee & Chuah, 2008; Yoo, Gujar, Hu, Jolesz, & Walker, 2007) has emerged showing that the brain structural or functional connectivity (FC) are influenced by acute sleep loss.

In particular, resting‐state functional MRI (rs‐fMRI) enables us to record the brain spontaneous activities and to measure their intrinsic FC among distinct brain regions. Relative to rested wakefulness (RW), some rs‐fMRI studies (Chen et al., 2018; Kaufmann et al., 2016; Samann et al., 2010; Tashjian, Goldenberg, Monti, & Galvan, 2018) revealed that the brain regions belonging to the default mode network (DMN) and subcortical network were more susceptible to worsen sleep quality. For instance, Shao et al. (2013) conducted a seed‐based analysis and found that FC strengths between the thalamus, which plays a critical role in arousal regulation, and several temporal and prefrontal regions were reduced after 36 hr of SD. Regarding the changes of the DMN subnetwork, Chen et al. (2018) found significantly decreased connectivity within the dorsal DMN but increases within the ventral DMN and between two subsystems after 24 hr of SD, as well as significant correlations between these changes and working memory ability and psychomotor vigilance task speed.

Recently, dynamic functional connectivity (dFC) approach (Allen et al., 2014; Calhoun, Miller, Pearlson, & Adali, 2014; Chang & Glover, 2010; Preti, Bolton, & Van De Ville, 2017) has been proposed to capture the temporal features of spontaneous brain activities via short frames of time courses during rs‐fMRI scanning. For dFC analysis, the covariances were calculated across entire sliding time windows of all participants and then clustered into several brain connectivity states. Next, the dFC properties were used for investigating their time‐varying abnormalities in specific populations compared to healthy controls (Damaraju et al., 2014; Diez‐Cirarda et al., 2018; Dong et al., 2018; Rashid et al., 2018). For example, Rashid et al. (2018) observed that a high level of autistic traits and autism spectrum disorder diagnosis was closely associated with a globally disconnected FC pattern in 774 6‐ to 10‐year old children. Moreover, these dFC characteristics achieved high accuracy rate in distinguishing various diseases (Jin et al., 2017; Yao et al., 2017). Comparing 36 hr of SD with RW, Xu et al. (2018) identified different occurrence probabilities of seven SD‐ and RW‐dominant connectivity states. Using an index of eyelid closure in sleep‐deprived subjects, Wang et al., (2016) estimated two connectivity states reflecting FC pattern of high‐ and low‐arousal levels. The occurrence of these states predicted inter‐subjects' behavioral lapsing and intra‐subjects' response speeds (Patanaik et al., 2018; Yeo, Tandi, & Chee, 2015). However, several studies (Choe et al., 2017; Zhang, Baum, Adduru, Biswal, & Michael, 2018) debated the low replicabilities of both temporal brain fluctuations and their dFC parameters across sliding windows using some large samples of public datasets.

Therefore, we hypothesized that: (a) the dFC properties drawn from the temporal features of spontaneous brain activities have high test–retest reliability between two separate RW sessions, (b) the dFC matrices of some specific brain connectivity states could effectively reflect the altered temporal features after acute SD, and (c) the dFC instabilities correlate with the deficiencies of neuropsychological performances.

To answer these questions, we performed two independent within‐subjects laboratory studies with rs‐fMRI scanning and neuropsychological tests after SD and during RW/RS conditions. In details, we first evaluated the test‐retest reliability of dFC properties during two separate sessions of RW conditions prior to the 28 hr of SD (Study 1). Then, we statistically examined the differences of dFC properties after 28 hr of SD to two separate sessions of RW conditions (Study 1). Similarly, we compared FC during wakefulness after 52 hr of SD and after 14 hr of recovery sleep (Study 2). Furthermore, we performed correlation analysis between dFC parameters of the brain states and neuropsychological tests. Additionally, we examined the similarities of the estimated brain states between two studies by combing their rs‐fMRI datasets. Finally, to ensure robustness of our main results, we tested out different sliding window sizes and numbers of clusters prior to the final analysis.

## 2 METHOD AND MATERIALS

### 2.1 Participants and study design

A prior statistical power analysis was conducted based on the results of a similar previous study comparing dFC properties in healthy controls after 36 hr of SD and RW (Xu et al., 2018). The effect size in this study was 1.68, which can be considered to be extremely large using Cohen's (1988) criteria. Based on an alpha = .05 and a power = 0.80, the projected sample size needed with this effect size (GPower 3.1.9.4 software) is approximately *N* = 6 to detect significant effects between SD and RW/recovery sleep (RS). Thus, our proposed sample size of 13/14 will be sufficient for the purpose to detect the expected between‐conditions differences.

#### 2.1.1 SD28 study

Fifteen healthy young volunteers (seven female; age: 27.8 ± 5.57 years; mean body mass index: 23.59 ± 2.91) were recruited in this study. Two participants were excluded because of the inconsistent scanning parameters (different Time of Repetition) of rs‐fMRI datasets. All subjects underwent clinical examinations to exclude any psychiatric or neurological diseases. One week before arriving to the sleep research lab, the participants were instructed to maintain 9 hr of sleep duration per day (22:00/23:00 hr–7:00/8:00 hr) for sleep satiation that was verified by actigraphy. Once they came to the lab, the sleep episodes were scheduled from 23:00 to 7:00 hr or from 00:00 to 8:00 hr for nine consecutive days, then participants stayed awake for 36 hr. Afterward, all participants ended with a final 14‐hr RS episode (21:00–7:00 hr or 22:00–8:00 hr). The participants were allowed to do some activities and continuously monitored by our research staff. Light level was measured at ~650 lx. Participants were nonsmokers and instructed to refrain from caffeine (confirmed by self‐determination) and alcohol intake 1 week before and during the study. Two days prior and 1 day prior to SD, we performed MRI scans, labeled as RW1 and RW2 in this study. The two scans were scheduled 24 hr apart and were carried out either at 11:00 hr or at 13:00 hr. The third MRI scan was taken 24 hr after RW2 at 28/29 hr of SD (SD28). Before each MRI scan, we conducted a 10‐min version of a psychomotor vigilance task (PVT; Dinges & Powell, 1985), both a spatial 3‐back task and a letter 3‐back task, and the Karolinska Sleepiness Scale (KSS; Akerstedt & Gillberg, 1990). The averaged PVT‐lapses (reactions longer than 500 ms), PVT‐speed, n‐back hits, and KSS sleepiness scores were computed to assess participants' level of sustained attention, working memory, and subjective sleepiness (more detailed are provided in Supporting Information). This study was approved by the Ethics Committee of the Ärztekammer Nordrhein and informed consent was obtained from all participants.

#### 2.1.2 SD52 study

In this study, we enrolled 14 young males (age: 28.21 ± 5.21 years, mean body mass index: 24.39 ± 3.58). Study procedures and neuropsychological tests were documented in detail in a previous publication (Elmenhorst et al., 2017). In brief, the participants were requested to maintain their regular sleep habits (time in bed: 23:00 hr to 7:00 hr), to refrain from caffeine and wear a wrist‐actigraph during 1 week, 5 days, and 3 days before coming to the sleep research lab, respectively. MRI scans and neuropsychological tests were completed after 52 hr of SD (SD52) and 14 hr of RS (RS14). Light condition and other setups were as same as those of the SD28 study. The MRI scanning time was same as the SD28 study, that is, either at 11:00 hr or at 13:00 hr. The neuropsychological tests in this study included a 3‐min version of PVT, spatial 3‐back task, and KSS sleepiness scores. This study was approved by the Ethics Committee of the Medical Faculty of the University of Düsseldorf and informed consent was obtained from all participants.

### 2.2 MRI acquisitions

We used a 3T MR‐PET Scanner (Biograph mMR, Siemens) to collect the MRI datasets for SD28 study. During the rs‐fMRI scanning, each participant was instructed to keep the eyes open and concentrate on the cross presented by a MRI‐compatible screen inside the scanner room. Additionally, a camera was equipped to monitor the participants' wakefulness during scanning. If the eyes of participants were closed longer than a blink, they would be addressed over the intercom system up by the research staff. To diminish the occurrences of microsleep inside the scanner, we only acquired ~5 min of resting‐state fMRI datasets for each run of subject. Under this condition, their neuronal fluctuations were identified as high wake probability thus they were more likely to stay awake (Tagliazucchi & Laufs, 2014). More specifically, rs‐fMRI datasets were acquired using a gradient‐echo echo planar imaging (GE‐EPI) sequence with following parameters: Time of Repetition (TR) = 2.3 s, Echo Time (TE) = 30 ms, Flip angle = 90°, matrix size = 64 * 64, number of slices = 36, slice thickness = 3.1 mm, voxel size = 3.1 * 3.1 mm^2^, 146 volumes in total. Meanwhile, we conducted a 3D magnetization‐prepared rapid acquisition gradient echo (MPRAGE) anatomical scanning with following parameters: TR = 2.25 s, TE = 3.03 ms, matrix size = 256 * 256, number of slices = 176, voxel size = 1 * 1 mm^2^, slice thickness = 2.25 mm, flip angle = 90°. For the SD52 study, we used a 3T Siemens MAGNETOM Trio MRI scanner (Erlangen, Germany) with a 32‐channel head coil to collect the MRI datasets using same parameters except TR = 2.2 s and 165 volumes for the rs‐fMRI scanning.

### 2.3 Rs‐fMRI preprocessing

We preprocessed the rs‐fMRI datasets using DPABI_V3.1 (http://rfmri.org/dpabi; Yan, Wang, Zuo, & Zang, 2016) which ran in SPM12 (Revision Number: 7219, http://www.fil.ion.ucl.ac.uk/spm/software/spm12/). Briefly, the first six and five volumes were discarded to make sure the participants were adapted to the MRI environments for SD28 and SD52 study, respectively. Then, we conducted slice‐timing correction and realignment for slices acquisitions and head motion corrections. Subjects were not excluded as they did not exceed the head transitions <3 mm, rotations <3° or the mean framewise displacement (FD) value <0.6 mm (Power, Barnes, Snyder, Schlaggar, & Petersen, 2012). As a result, the mean FD values were calculated (SD28: RW1, 0.17 ± 0.10 mm; RW2, 0.14 ± 0.05 mm; SD28, 0.21 ± 0.16 mm; SD52: SD52, 0.16 ± 0.06 mm; RS14, 0.15 ± 0.07 mm) and no subjects were excluded in further analysis. The Friston 24‐motion parameters, signals from cerebrospinal fluid and white matter were regressed out as the nuisance variables. Finally, we normalized the preprocessed fMRI images to the MNI standard template space using the DARTEL algorithm and smoothed them using 4 mm full width at half maximum. As several studies (Wong, Olafsson, Tal, & Liu, 2013; Yeo et al., 2015) evidenced that the global signal fluctuations often co‐occurred with the arousal drops, we did not perform the global signal regression during the pre‐processing steps. Prior to independent component analysis (ICA), the voxel‐wise variance normalization (*z*‐scored) was conducted for optimizing the following temporal decomposition (Allen et al., 2014).

### 2.4 ICA and preprocessing

To reproduce the independent components (ICs) for our two SD studies, we separately employed Group ICA of fMRI Toolbox (GIFTv3.0b, http://mialab.mrn.org/software/gift/) to estimate the ICs and postprocess our ICA results. First, we reduced the subject‐specific datasets to 120 ICs using principal components analysis (PCA) algorithm and constrained the group datasets to 100 ICs using expectation–maximization (EM) algorithm (Roweis, 1998). To replicate our decomposed ICs, the Infomax ICA algorithm was repeated for 10 times in ICASSO and the aggregate spatial maps (SMs) were generated. Using the group ICA back reconstruction method (Erhardt et al., 2011), we then generated the subject‐specific SMs and their corresponding time courses. We identified ICs if their peak activations were located in gray matter and further categorized them into following subnetworks: Subcortical (SC), auditory (AUD), default‐mode network (DMN), visual (VIS), somatomotor (SM), cognitive control (CC), and cerebellar (CB) networks. Finally, we postprocessed the ICA results with detrending linear, quadratic, and cubic trends, removal of detected outliers, and low‐pass filtering with a frequency cutoff of 0.15 Hz.

### 2.5 Dynamic FC processing

The dFC approach was conducted with Temporal dynFN toolbox implemented in the GIFTv3.0b software. We applied a sliding window of 22 TRs (50.6 s and 48.4 s, respectively) with a Gaussian alpha value (σ = 3TRs) and a step between windows of 1TR to divide the time course resulting in 118 and 138 sliding‐windows, respectively. To reduce the inadequacies of short sliding time windows, we estimated variances from the regularized precision matrix (inverse covariance matrix; Smith et al., 2011). Based on all covariance matrices, the number of clusters (*k*) was determined by the elbow criterion of cluster validity index and cluster centroids of brain states were calculated by *k*‐means clustering algorithm. Consequently, we estimate the dFC properties including number of transitions, mean dwell time, and fraction of states. Briefly, number of state transitions refers to the number of times that a brain state changes into another state for each subject. Mean dwell time is measured by the number of total sliding windows of one brain state divided by the number of transitions entering this state. Accordingly, the fraction of state represents the percentage of the number of sliding windows spent in one state of total number of sliding windows.

### 2.6 Statistical comparisons and correlations analysis

For the test–retest reliability of each dFC parameter, the intraclass correlation coefficient (ICC) and their 95% confidence intervals for two separate sessions of RW conditions were calculated based on a single‐rating, absolute‐agreement, two‐way mixed‐effects model which is implanted in SPSS22. Subsequently, we respectively applied one‐way repeated ANOVA analysis (factor condition: RW1, RW2, SD28; or factor condition: SD52, RS14) and paired *t* test (two‐tailed) to examine the statistical significances of their dFC properties and neuropsychological tests for the SD28 and SD52 studies. For the ANOVA analysis, Tukey's honestly significant difference was utilized to perform post hoc tests. Statistical power for each study was indicated by the effect size using Cohen's *d* (Hojat & Xu, 2004) and partial eta squared (η^2^). Additionally, we computed the Pearson's correlations coefficient between dFC parameters and neuropsychological tests for both, SD28 and SD52. All of above statistical comparisons and correlations were conducted using SPSS statistical package version 22 (SPSS Inc., Chicago, IL) at an uncorrected significant level of *α* < .05.

### 2.7 Validations of the altered dFC properties

Additionally, we processed the two datasets by using a different number of clusters (*k* = 4) or sliding‐window length (30 TRs) to investigate the robustness of the methods. Furthermore, both rs‐fMRI datasets were pooled together for an overall group ICA analysis in order to apply the same independent components and subnetworks of the two studies to the dFC analysis. Cross‐correlations of the dynamic connectivity states were computed to indicate their similarity. Lastly, we independently applied global signal regression (GSR) to access its influences on our main conclusions during the preprocessing procedures.

## 3 RESULTS

### 3.1 Differences on head motion and neuropsychological tests

Head motion (mean FD values) was not significantly different between conditions (SD28: F[2,11], *p* = .13, η^2^ = 0.16; SD52, *t* = 0.79, *p* = .44, Cohen's *d* = 0.21). For the SD28 study, we observed significant increases of PVT‐lapses (F[2,11] = 18.28, *p* < .001, η^2^ = 0.60) and KSS sleepiness scores (F[2,11] = 32.04, *p* < .001, η^2^ = 0.73), but significant reductions in PVT‐speed (F[2,11] = 22.42, *p* < .001, η^2^ = 0.65) and spatial 3‐back hits (F[2,11] = 14.62, *p* < .001, η^2^ = 0.55) after 28 hr of SD compared to RW1 and RW2 (Table S1). For the SD52 study, we found significantly increased PVT‐lapses (*t* = 2.5, *p* = .026, Cohen's *d* = 0.78), and KSS sleepiness scores (*t* = 14.27, *p* < .001, Cohen's *d* = 4.96), and decreased PVT‐speed (*t* = −5.68, *p* < .001, Cohen's *d* = 1.52) after 52 hr of SD compared to 14 hr of RS (Table S2).

### 3.2 Test–retest reliability of dFC properties during two sessions of RW

**Figure 1:**
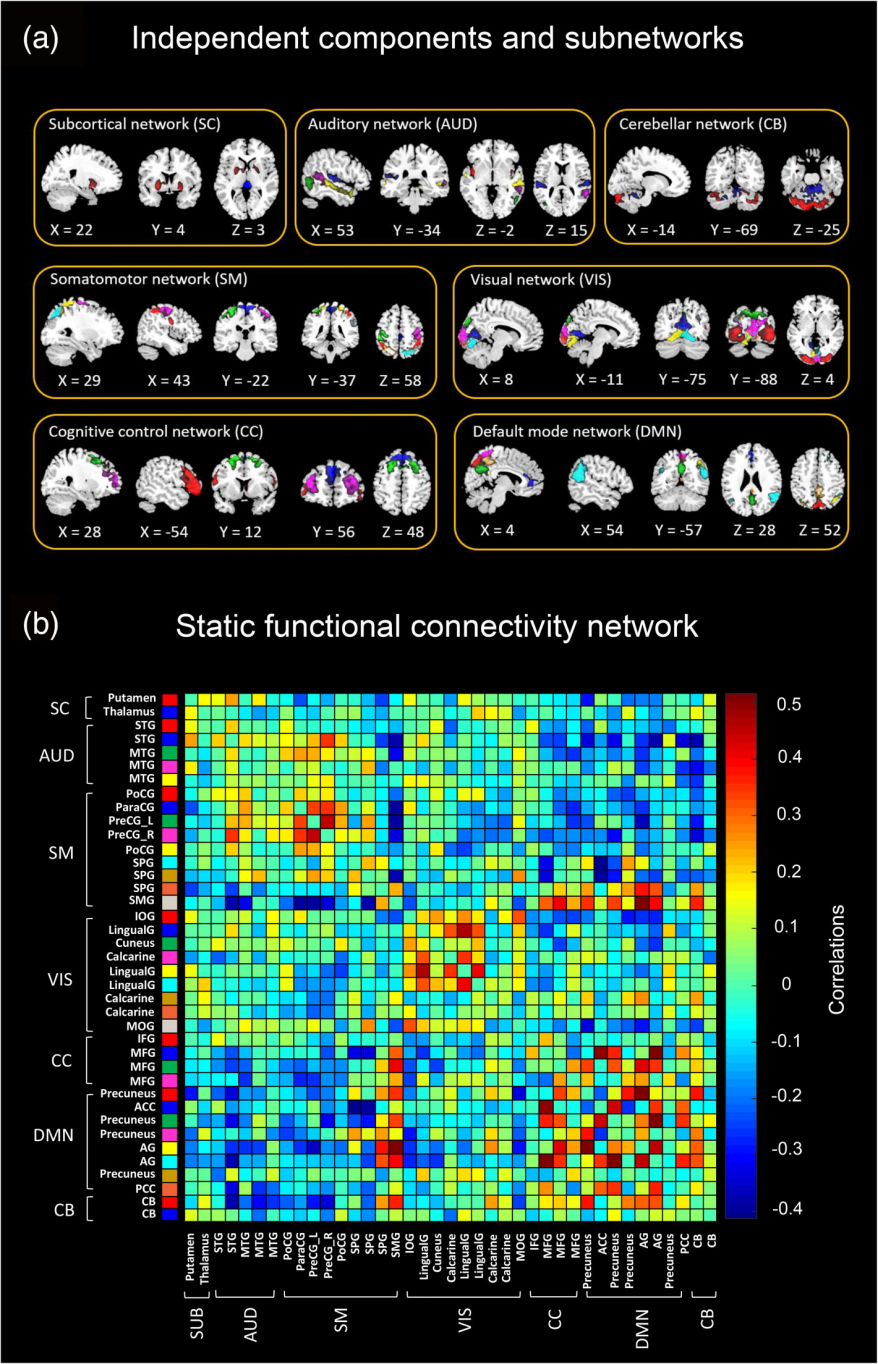
Independent functional components and corresponding functional connectivity network for the 28 hr of sleep deprivation study. Abbreviations: ACC, anterior cingulate cortex; AG, angular gyrus; CB, cerebellar; IOG, inferior occipital gyrus; IFG, inferior frontal gyrus; LingualG, lingual gyrus; MFG, middle frontal gyrus; MOG, middle occipital gyrus; MTG, middle temporal gyrus; ParaCG, paracentral gyrus; PoCG, postcentral gyrus; PreCG, precentral gyrus; PCC, posterior cingulate cortex; SPG, superior parietal gyrus; SMG, supramarginal gyrus; STG, superior temporal gyrus

**Figure 2:**
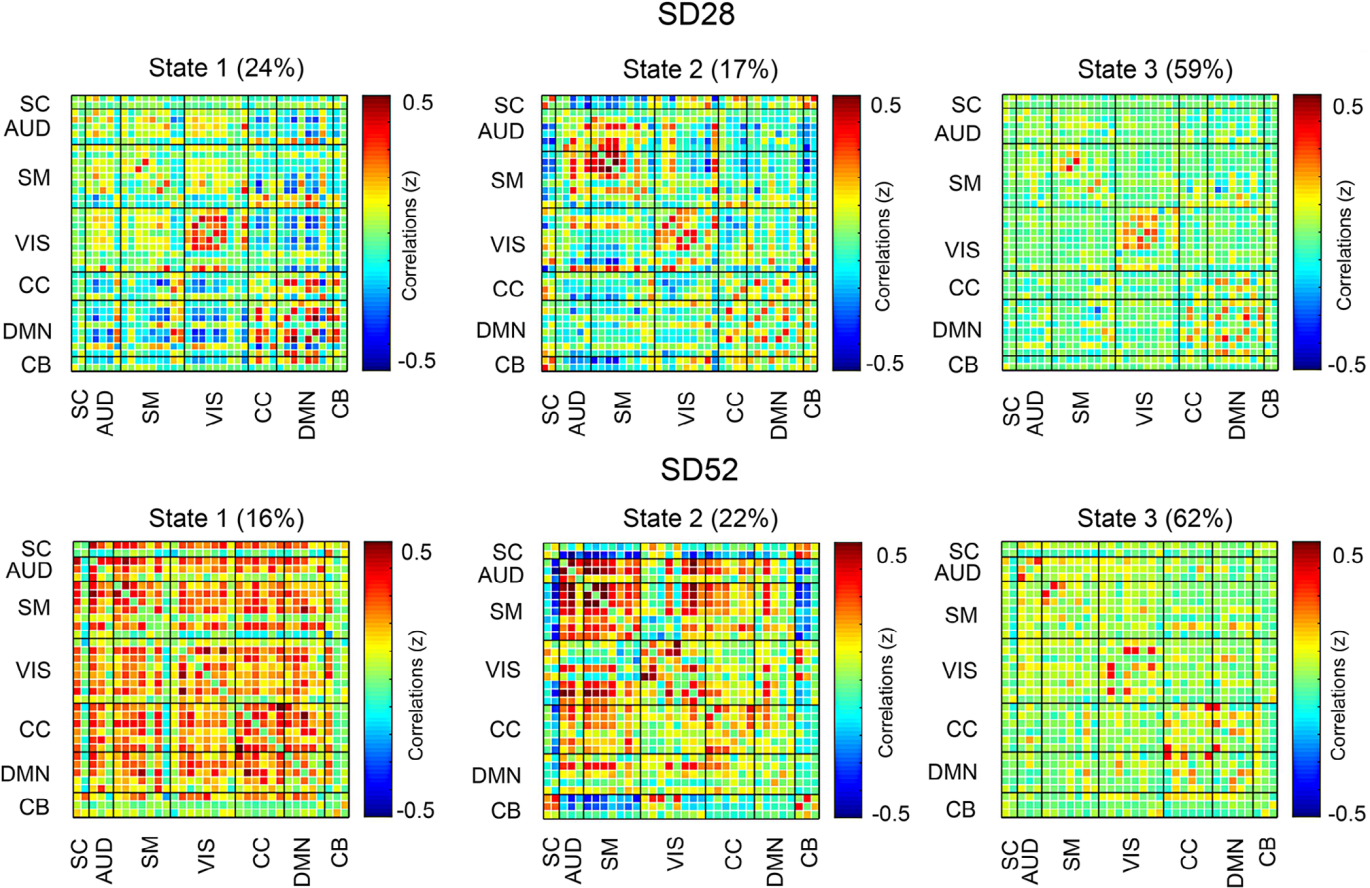
Cluster centroids of brain states for 28 hr and 52 hr of sleep deprivation. The percentage means the occurrence probability of the specific brain state across the sliding windows of all subjects. Abbreviations: AUD, auditory network; CB, cerebellar network; CC, cognitive control network; DMN, default mode network; SC, subcortical network; SM, somatomotor network; VIS, visual network

Figure 1 illustrated our identified 39 ICs for the SD28 study and these ICs were categorized into seven functional subnetworks (Figure 1). Notably, mostly ICs derived from the SD28 study were replicated through our SD52 study (37 of total 39 ICs, Figure S1). According to the elbow criterion, the number of clusters was determined as 3. Subsequently, we estimated the corresponding cluster centroids of brain connectivity states (Figure 2), as well as their dFC parameters. For the test–retest reliability of RW (Table 1), we found that the number of state transitions showed a lower ICC value compared to both the mean dwell time and fraction of states. Moreover, this finding was reproduced when we set the sliding‐window length = 30 TRs or *k* = 4, respectively (Table 1).

**Table 1:** Test–retest reliability (TRT), which is indicated by the intraclass correlation coefficient (ICC) and their 95% confident intervals, of the dynamic functional connectivity parameters during two rested wakefulness conditions in the 28 hr of sleep deprivation study

		Mean dwell time (95% CI)	Fraction of states (95% CI)	Number of state transitions (95% CI)
22 TRs, *k* = 3	State 1	0.64 (0.18, 0.87)	0.78 (0.41, 0.93)	0.29 (0.13, 0.65)
	State 2	0.39 (−0.12, 0.76)	0.59 (0.11, 0.85)	
	State 3	0.61 (0.09, 0.86)	0.94 (0.82, 0.98)	
22 TRs, *k* = 4	State 1	0.63 (0.16, 0.87)	0.89 (0.68, 0.96)	0.68 (0.05, 0.90)
	State 2	0.71 (0.28, 0.9)	0.98 (0.92, 0.99)	
	State 3	−0.06 (−0.52, 0.47)	−0.03 (−0.51, 0.49)	
	State 4	0.81 (0.49,0.94)	0.96 (0.88, 0.99)	
30 TRs, *k* = 3	State 1	0.86 (0.61, 0.95)	0.82 (0.52, 0.94)	0.36 (0.09, 0.81)
	State 2	0.42 (−0.12, 0.78)	0.604 (0.13, 0.86)	
	State 3	0.55 (0.004, 0.84)	0.83 (0.52, 0.94)	

*Note*: The first column represents the selected sliding window and number of clusters for dynamic functional connectivity analysis. Abbreviations: CI, confidence interval; *k*, number of clusters; TRs, time of repetitions.

### 3.3 Altered dFC properties after acute SD

**Figure 3:**
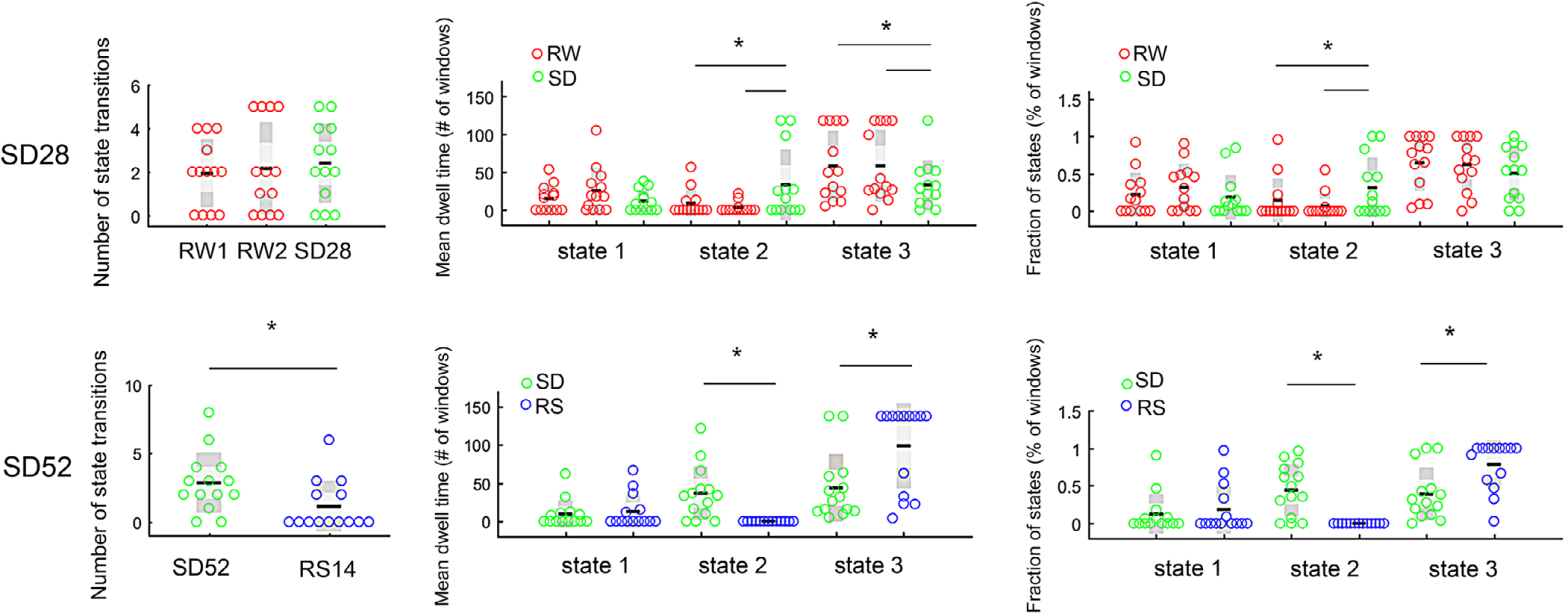
Statistical comparisons of dynamic functional connectivity during rested wakefulness (RW), sleep deprivation (SD) and following recovery sleep (RS). Number of subjects = 13 and 14 in SD28 and SD52 study. Black line indicates the mean value of each group and light gray area represents the 95% confidence interval. * Statistically different at *p* < .05 level

For the SD28 study, there was no significant difference in number of state transitions (F[2,11] = 0.25, *p* = .78, η^2^ = 0.02) among the three conditions (i.e., RW1, RW2, and SD28). Meanwhile, we found the mean dwell time and fraction of State 2 were significantly increased (F(2,11) = 5.63, *p* = .03, η^2^ = 0.32; F(2,11) = 4.69, *p* = .02, η^2^ = 0.28), but mean dwell time of State 3 was significantly reduced (F[2,11] = 3.78, *p* = .04, η^2^ = 0.24) after SD (Figure 3). Follow‐up comparisons indicated that each pairwise comparison between the 28 hr of SD and RW was significantly different. The dFC parameters of State 1 did not show significant differences among three conditions (Figure 3).

For the SD52 study, we also estimated the cluster centroids of three brain states when *k* = 3 and found that number of state transitions missed significance (*t* = 2.05, *p* = .061, Cohen's *d* = 0.81) after SD relative to after 14 hr of RS (Figure 3). Interestingly, the brain State 2 was only detected in the sleep‐deprived conditions, but both mean dwell time and fraction of State 3 were significantly reduced (*t* = −3.13, *p* = .008, Cohen's *d* = 0.84; *t* = −3.68, *p* = .003, Cohen's *d* = 0.98; Figure 3).

### 3.4 Correlations analysis after acute SD

**Figure 4:**
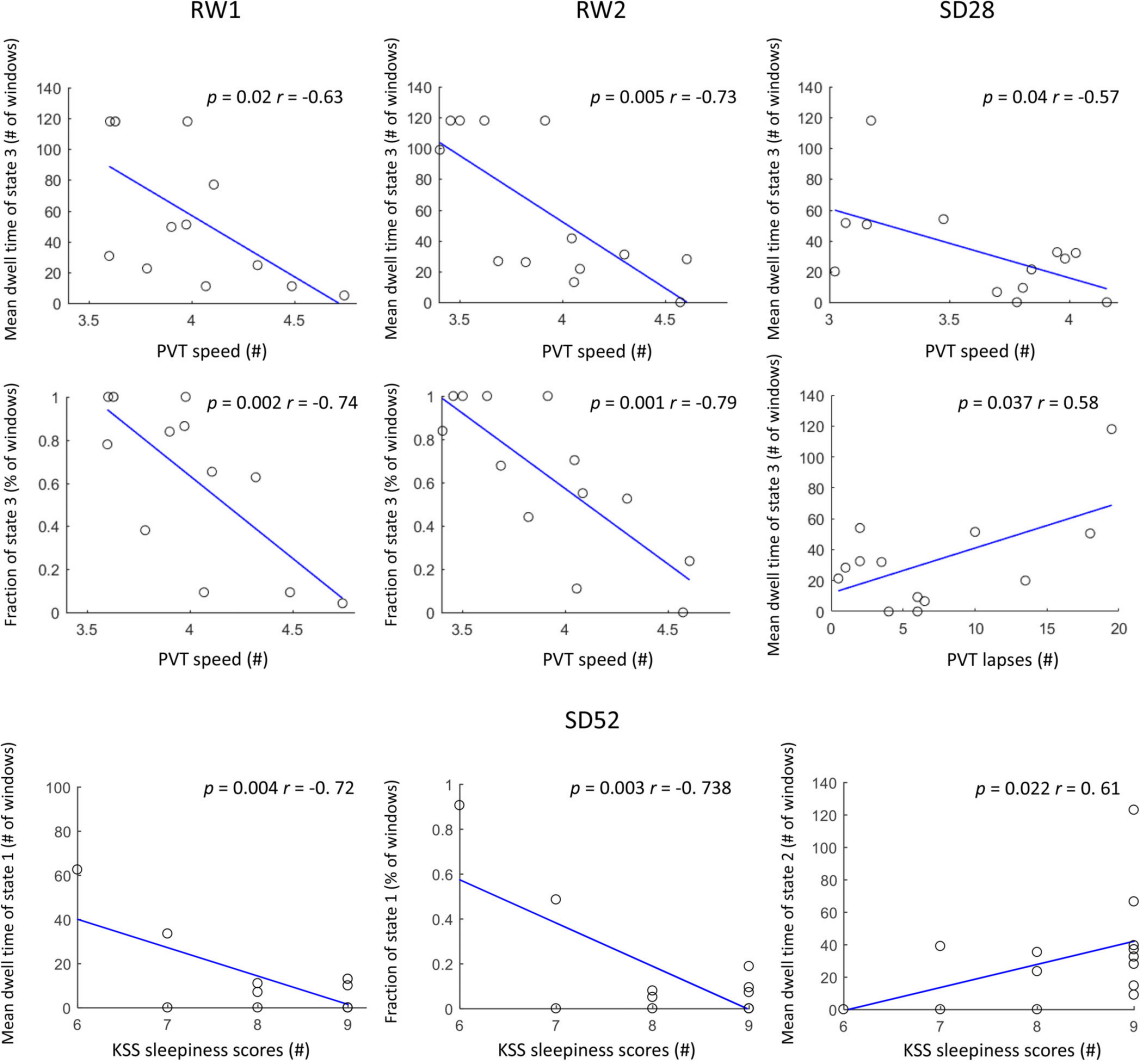
Correlations between dynamic functional connectivity properties and neuropsychological test scores. Number of subjects = 13 and 14 in SD28 and SD52 study. Abbreviations: KSS, Karolinska Sleepiness Scale; PVT, psychomotor vigilance task; RW, rested wakefulness; SD28, 28 hr of sleep deprivation; SD52, 52 hr of sleep deprivation. The *r* values represent Pearson correlation coefficients

**Figure 5:**
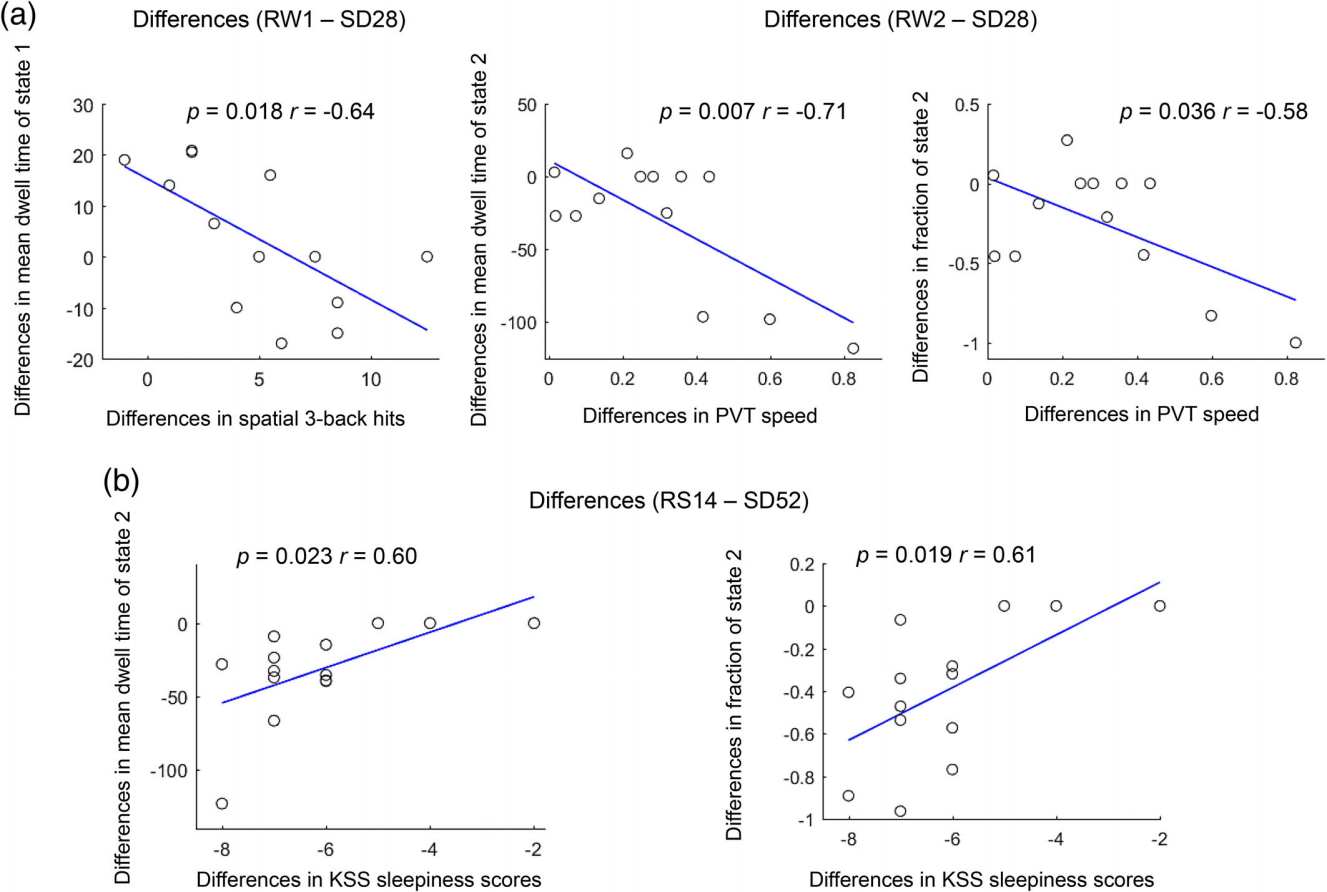
Correlations of the differences (A, RW1‐SD28; B, RW2‐SD28; C, RS14‐SD52) of dynamic functional connectivity properties and neuropsychological tests. Number of subjects = 13 and 14 in SD28 and SD52 study

For the SD28 study, our correlations revealed that the increased mean dwell time and fraction of State 3 was significantly associated with a decreased PVT‐speed during both RW1 (Figure 4, *p* = .022, *r* = −.63; *p* = .004, *r* = −.74) and RW2 (*p* = .005, *r* = −.73; *p* = .001, *r* = −.79). After 28 hr of SD, an increase in mean dwell time of State 3 was also significantly correlated with a decrease in PVT‐speed (*p* = .044, *r* = −.57) and an increase in PVT‐lapses (*p* = .038, *r* = .56). Meanwhile, an increase in mean dwell time and fraction of State 2 under SD relative to RW (RW1‐SD28 and RW2‐SD28) was significantly correlated with a decrease in spatial 3‐back hits (Figure 5a, *p* = .018, *r* = −.64) and PVT‐speed (*p* = .007, *r* = −.71; *p* = .036, *r* = −.58). We did not observe any significant correlations between other brain states and the neuropsychological tests.

For the SD52 study, the increases of mean dwell time and fraction of State 1 was significantly associated with a decrease in KSS sleepiness scores (Figure 4, *p* = .004, *r* = −.72; *p* = .003, *r* = −.738) but an increase in the fraction of State 2 was significantly correlated with an increase in sleepiness (*p* = .022, *r* = .61) after 52 hr of SD. Meanwhile, the increases in mean dwell time and fraction of State 2 under SD relative to recovery conditions (RS14‐SD52) was also significantly correlated with an increase in KSS sleepiness scores (Figure 5b, *p* = .023, *r* = .60; *p* = .019, *r* = .61). We did not find any significant associations between other brain states and the neuropsychological tests.

### 3.5 Altered dFC properties after acute SD

Similar results were replicated for the SD28 and SD52 studies when applying a sliding‐window length = 30 TRs or *k* = 4 (please refer to Figures S2, S3, S5, and S6 for more details). Meanwhile, the use of GSR reproduced our main findings partly due to the fact of significantly decreased dynamic properties of State 2 but decreases of State 3 (datasets not shown). For the joint rs‐fMRI analysis, we identified 42 independent components and estimated the corresponding connectivity states. Notably, we found State 2 and State 3 of SD28 were significantly correlated with the States 2 and 3 of SD 52 (Figure S4, *r* = .36 and *r* = .51), as well as the between‐group differences of dFC metrics after SD (Figure S6).

## 4 DISCUSSION

In this current study, we applied a dynamic approach to investigate the alterations of time‐varying fluctuations of FC after SD. We observed that mean dwell time and fraction of two brain connectivity states were significantly altered after acute SD relative to either the RW or RS condition. One of these connectivity states (State 2) was significant increased after prolonged SD, which might represents a manifestation of light sleep/drowsiness. Moreover, this state was only present after 52 hr of SD in comparisons with 14 hr of RS. On the contrary, the dFC parameters (mean dwell time and fraction) of State 3 that occupied high proportion across entire sliding windows were remarkably reduced after acute SD in our two SD studies. Moreover, the test–retest reliability of dFC properties across two wakefulness states suggested that mean dwell time and fraction of states are two highly reliable measures for evaluating the time‐varying fluctuations of spontaneous brain activities. Finally, we validated the dFC characteristics of the identified brain states and their sleep‐loss related alterations were reproducible using a different number of clusters or sliding window lengths. Using the same independent components and subnetworks, we further found high similarities of State 2 and State 3 between two studies, as well as their changes after sleep loss. In summary, these results greatly enhance our understanding on the temporal features of dFC characteristics after acute SD and are promising to assess the individual's arousal/sleepiness level.

We found that 52 hr of SD induced a higher frequency of state transitions. This finding is similar to prior work (Xu et al., 2018) that discovered higher transition probabilities among SD‐dominant states after 36 hr of SD. Thus, these results suggest that extended wakefulness can indeed accelerate the shifts between distinct connectivity states across all participants. Nevertheless, it should be noticed that this effect was not present after 28 hr of SD that may be due to the duration of time awake.

The time spent in brain State 2 was significantly increased after acute SD and correlated with high sleepiness. The cluster centroids of this connectivity state are illustrated by strong anti‐correlated functional connections between subcortical and task‐positive cortical subnetworks, and hyper‐activated FC couplings within task‐specific subnetworks. Previous sleep‐related FC studies (Killgore et al., 2015; Picchioni et al., 2014; Shao et al., 2013) evidenced that prolonged wakefulness deactivated or even inhibited thalamocortical connections, deteriorating alertness and processing of sensory or visual information. In fact, using sliding‐window correlations, a comparable connectivity state was repetitively estimated and proposed as the FC configuration of light sleep/drowsiness (Abrol et al., 2017; Damaraju et al., 2014). Furthermore, in a simultaneous EEG‐fMRI study (Allen, Damaraju, Eichele, Wu, & Calhoun, 2018), the FC characteristics of this connectivity state were correlated with reduced EEG alpha but increased delta and theta power reflecting drowsiness which has been shown repeatedly in EEG experiments (Aeschbach et al., 1999). Regarding the influences of acute sleep loss, Xu et al. (2018) identified three comparable SD‐dominant states with increased dwell time and transition probabilities in 37 young males after 36 hr of SD. Meanwhile, a series of dFC studies (Patanaik et al., 2018; Wang et al., 2016; Yeo et al., 2015) assessed two discrete connectivity states representing brain activities of high and low arousal levels of which the latter one was accompanied by an impairment of cognitive performance especially with respect to PVT‐lapses, speed of PVT processing and 3‐back working memory. Additionally, during an auditory vigilance task (AVT)‐based fMRI scanning, the AVT‐lapses were significantly positively and negatively correlated with low arousal and high arousal states, respectively (Wang et al., 2016). In our studies, the increased occurrence of State 2 under SD condition was associated with a decrease in PVT response speed and an increase in KSS sleepiness scores. Therefore, our results provide evidence that this connectivity state reflects the neural substrate of light sleep/drowsiness. More importantly, its increases may represent a potential biomarker for daytime sleepiness and compromised cognitive performance healthy individuals.

Connectivity State 3 was predominant (59 and 62%) for both the SD28 and SD52 studies. This globally hypo‐connected FC network is featured with higher FC strengths within subnetworks than inter‐subnetwork connections. To our knowledge, similar characteristics of this state and its high proportion were also observed by other dFC studies (Diez‐Cirarda et al., 2018; Marusak et al., 2017; Rashid et al., 2018). Allen et al. (2014) attributed this state to the average of large numbers of extra connectivity states that were not adequately distinct or frequent to be separated. Yet, the increased mean dwell time of a comparable state was significantly positive correlated with self‐focused thoughts in a population of healthy children (Marusak et al., 2017). Rashid et al. (2018) demonstrated this state's close associations with high levels of autistic traits and autism spectrum disorder diagnosis. Giving these outcomes, we assume that this connectivity state is primarily involved with the self‐consciousness processing. In our studies, the reduction of occurrence probability in connectivity State 3 might be a consequence of disabled retaining of the self‐relative mental activities after a lack of sleep. Furthermore, reduced occurrence of this state was associated with slower PVT response speed and more PVT lapses. Together with the changes of State 2, we propose that, after prolonged wakefulness, State 3 is substituted by increasing occurrences of brain connectivity state reflecting light sleep/drowsiness, and as a result, induce higher number of state transitions between connectivity states.

Notably, there are some additional or uncontrolled factors that could have influenced our results in these two SD studies, such as a difference in sex distribution, duration of prolonged wakefulness, and the circadian phase. Possibly, owing to these factors, we detected that the FC profile of Connectivity State 1 in the SD28 study is characterized by strong positive connections within DMN and negative correlations between DMN and other cortical subnetworks. In contrast, the network architecture of Connectivity State 1 in the SD52 study is featured by the extensive hyperconnected connections. Further, statistical comparisons of dFC properties in these states exhibited no significant differences between the acute SD and RW/RS conditions suggesting the occurrence probability of this state is less influenced by prolonged wakefulness. Nevertheless, after 52 hr of sleep loss, the significant correlations between the occurrence probabilities of Connectivity State 1 and KSS sleepiness scores indicate that it probably represents whole brain FC configuration of high arousal level. Consistent with other states, our results were replicated across different sliding‐window length and number of clusters.

Our test–retest evaluation of dFC properties revealed that the number of state transitions is a less reliable parameter. On the other hand, our findings highlight the importance of mean dwell time and fraction of connectivity states potentially acting as robust statistical indicators with low intra‐subject variabilities. Up to now, there have been several studies that attempted to examine the test–retest reliability of dynamic correlations and their connectivity states, as well as the summary measures of states (Abrol et al., 2017; Choe et al., 2017). For instance, Zhang et al., 2018 analyzed the reliability of both static FC and dynamic fluctuations (standard deviation, amplitude of low frequency fluctuations, and excursions) in a large population of adults (820 subjects from Human Connectome Project). They found the variations of connectivity dynamics were most robust when the sliding‐window length was less than 40 s. However, Choe et al. (2017) found, although the temporal features of connectivity dynamics were reliably reproducible, their dFC parameters of connectivity states (number of state transitions and dwell time) had poorer reliabilities across the sliding windows, taped sliding window, and dynamic conditional correlations approach. Thus, our results are informative to demonstrate the robustness of dFC properties in terms of their reproducibility during RW conditions across numbers of clusters or sliding‐window lengths.

Additionally, several issues should be taken into account in our study. First, the sample size was relatively small to evaluate the dFC abnormalities. To address this limitation, we analyzed two independent studies with the same data‐processing pipeline and examined their dFC alternations gaining similar results. Nevertheless, more participants should be recruited in the further and our study is exploratory. Second, although we demonstrated high reproducibility of our two brain states and their alterations across many parametric choices (sliding‐window length, number of clusters, and GSR), we acknowledge that the additional methodological approaches (e.g., a narrower frequency band filtering or the mathematical approaches of constructing correlation matrix) also have been proposed to remove nonneuronal noises or quantify variations of neuronal fluctuations (Leonardi & Van De Ville, 2015). In addition, it would be valuable to combine the FC approach with simultaneous EEG measurements to quantify the arousal level during rs‐fMRI scanning via EEG.

## 5 CONCLUSION

Using a sliding‐window correlations approach, we demonstrated that the prolonged wakefulness remarkably reduced the occurrences of a globally hypoconnected state reflecting self‐focused processing but increased amounts of a FC pattern representing light sleep/drowsiness. Moreover, sleep‐loss induced alterations of connectivity states were associated with increased sleepiness and impaired cognitive performance. Taken together, the results facilitate our knowledge in understanding how the specific brain states and their corresponding dFC characteristics are affected by sleep loss levels and offer potential biomarkers to reflect the individual's vulnerability.

## CONFLICT OF INTERESTS

We declaim there are no conflicts of interests.

## Supporting information


**Table S1** Statistical comparisons of neuropsychological variables among two sessions of rested wakefulness and 28 hr of sleep deprivation.

## Data Availability

The data that support the findings of this study are available from the corresponding author upon reasonable request.

## References

[hbm24855-bib-0001] Abrol, A. , Damaraju, E. , Miller, R. L. , Stephen, J. M. , Claus, E. D. , Mayer, A. R. , & Calhoun, V. D. (2017). Replicability of time‐varying connectivity patterns in large resting state fMRI samples. NeuroImage, 163, 160–176. 10.1016/j.neuroimage.2017.09.020 28916181 PMC5775892

[hbm24855-bib-0002] Aeschbach, D. , Matthews, J. R. , Postolache, T. T. , Jackson, M. A. , Giesen, H. A. , & Wehr, T. A. (1999). Two circadian rhythms in the human electroencephalogram during wakefulness. The American Journal of Physiology, 277(6), R1771–R1779. 10.1152/ajpregu.1999.277.6.R1771 10600925

[hbm24855-bib-0003] Akerstedt, T. , & Gillberg, M. (1990). Subjective and objective sleepiness in the active individual. The International Journal of Neuroscience, 52(1–2), 29–37. 2265922 10.3109/00207459008994241

[hbm24855-bib-0004] Allen, E. A. , Damaraju, E. , Eichele, T. , Wu, L. , & Calhoun, V. D. (2018). EEG signatures of dynamic functional network connectivity states. Brain Topography, 31(1), 101–116. 10.1007/s10548-017-0546-2 28229308 PMC5568463

[hbm24855-bib-0005] Allen, E. A. , Damaraju, E. , Plis, S. M. , Erhardt, E. B. , Eichele, T. , & Calhoun, V. D. (2014). Tracking whole‐brain connectivity dynamics in the resting state. Cerebral Cortex, 24(3), 663–676. 10.1093/cercor/bhs352 23146964 PMC3920766

[hbm24855-bib-0006] Calhoun, V. D. , Miller, R. , Pearlson, G. , & Adali, T. (2014). The Chronnectome: Time‐varying connectivity networks as the next frontier in fMRI data discovery. Neuron, 84(2), 262–274. 10.1016/j.neuron.2014.10.015 25374354 PMC4372723

[hbm24855-bib-0007] Chang, C. , & Glover, G. H. (2010). Time‐frequency dynamics of resting‐state brain connectivity measured with fMRI. NeuroImage, 50(1), 81–98. 10.1016/j.neuroimage.2009.12.011 20006716 PMC2827259

[hbm24855-bib-0008] Chee, M. W. L. , & Chuah, L. Y. M. (2008). Functional neuroimaging insights into how sleep and sleep deprivation affect memory and cognition. Current Opinion in Neurology, 21(4), 417–423. 18607201 10.1097/WCO.0b013e3283052cf7

[hbm24855-bib-0009] Chen, W. H. , Chen, J. , Lin, X. , Li, P. , Shi, L. , Liu, J. J. , … Shi, J. (2018). Dissociable effects of sleep deprivation on functional connectivity in the dorsal and ventral default mode networks. Sleep Medicine, 50, 137–144. 10.1016/j.sleep.2018.05.040 30055480

[hbm24855-bib-0010] Choe, A. S. , Nebel, M. B. , Barber, A. D. , Cohen, J. R. , Xu, Y. , Pekar, J. J. , … Lindquist, M. A. (2017). Comparing test‐retest reliability of dynamic functional connectivity methods. NeuroImage, 158, 155–175. 10.1016/j.neuroimage.2017.07.005 28687517 PMC5614828

[hbm24855-bib-0011] Damaraju, E. , Allen, E. A. , Belger, A. , Ford, J. M. , McEwen, S. , Mathalon, D. H. , … Calhoun, V. D. (2014). Dynamic functional connectivity analysis reveals transient states of dysconnectivity in schizophrenia. Neuroimage‐Clinical, 5, 298–308. 10.1016/j.nicl.2014.07.003 25161896 PMC4141977

[hbm24855-bib-0012] Diez‐Cirarda, M. , Strafella, A. P. , Kim, J. , Pena, J. , Ojeda, N. , Cabrera‐Zubizarreta, A. , & Ibarretxe‐Bilbao, N. (2018). Dynamic functional connectivity in Parkinson's disease patients with mild cognitive impairment and normal cognition. Neuroimage Clinical, 17, 847–855. 10.1016/j.nicl.2017.12.013 29527489 PMC5842729

[hbm24855-bib-0013] Dinges, D. F. , & Powell, J. W. (1985). Microcomputer analyses of performance on a portable, simple visual Rt task during sustained operations. Behavior Research Methods Instruments & Computers, 17(6), 652–655. 10.3758/Bf03200977

[hbm24855-bib-0014] Dong, D. , Duan, M. , Wang, Y. , Zhang, X. , Jia, X. , Li, Y. , … Luo, C. (2018). Reconfiguration of dynamic functional connectivity in sensory and perceptual system in schizophrenia. Cerebral Cortex, 29, 3577–3589. 10.1093/cercor/bhy232 30272139

[hbm24855-bib-0015] Elmenhorst, D. , Elmenhorst, E. M. , Hennecke, E. , Kroll, T. , Matusch, A. , Aeschbach, D. , & Bauer, A. (2017). Recovery sleep after extended wakefulness restores elevated A1 adenosine receptor availability in the human brain. Proceedings of the National Academy of Sciences of the United States of America, 114(16), 4243–4248. 10.1073/pnas.1614677114 28373571 PMC5402442

[hbm24855-bib-0016] Erhardt, E. B. , Rachakonda, S. , Bedrick, E. J. , Allen, E. A. , Adali, T. , & Calhoun, V. D. (2011). Comparison of multi‐subject ICA methods for analysis of fMRI data. Human Brain Mapping, 32(12), 2075–2095. 10.1002/hbm.21170 21162045 PMC3117074

[hbm24855-bib-0017] Goel, N. , Rao, H. , Durmer, J. S. , & Dinges, D. F. (2009). Neurocognitive consequences of sleep deprivation. Seminars in Neurology, 29(4), 320–339. 10.1055/s-0029-1237117 19742409 PMC3564638

[hbm24855-bib-0018] Harrison, Y. , & Horne, J. A. (2000). The impact of sleep deprivation on decision making: A review. Journal of Experimental Psychology. Applied, 6(3), 236–249. 11014055 10.1037//1076-898x.6.3.236

[hbm24855-bib-0019] Hojat, M. , & Xu, G. (2004). A visitor's guide to effect sizes ‐ statistical significance versus practical (clinical) importance of research findings. Advances in Health Sciences Education, 9(3), 241–249. 10.1023/B:AHSE.0000038173.00909.f6 15316274

[hbm24855-bib-0020] Jin, C. F. , Jia, H. , Lanka, P. , Rangaprakash, D. , Li, L. J. , Liu, T. M. , … Deshpande, G. (2017). Dynamic brain connectivity is a better predictor of PTSD than static connectivity. Human Brain Mapping, 38(9), 4479–4496. 10.1002/hbm.23676 28603919 PMC6866943

[hbm24855-bib-0021] Kaufmann, T. , Elvsashagen, T. , Alnaes, D. , Zak, N. , Pedersen, P. O. , Norbom, L. B. , … Westlye, L. T. (2016). The brain functional connectome is robustly altered by lack of sleep. NeuroImage, 127, 324–332. 10.1016/j.neuroimage.2015.12.028 26712339 PMC6600874

[hbm24855-bib-0022] Killgore, W. D. S. , Vanuk, J. R. , Knight, S. A. , Markowski, S. M. , Pisner, D. , Shane, B. , … Alkozei, A. (2015). Daytime sleepiness is associated with altered resting thalamocortical connectivity. Neuroreport, 26(13), 779–784. 10.1097/Wnr.0000000000000418 26177337

[hbm24855-bib-0023] Leonardi, N. , & Van De Ville, D. (2015). On spurious and real fluctuations of dynamic functional connectivity during rest. NeuroImage, 104, 464–465. 10.1016/j.neuroimage.2014.10.045 25234118

[hbm24855-bib-0024] Louca, M. , & Short, M. A. (2014). The effect of one night's sleep deprivation on adolescent neurobehavioral performance. Sleep, 37(11), 1799–1807. 10.5665/sleep.4174 25364075 PMC4196063

[hbm24855-bib-0025] Marusak, H. A. , Calhoun, V. D. , Brown, S. , Crespo, L. M. , Sala‐Hamrick, K. , Gotlib, I. H. , & Thomason, M. E. (2017). Dynamic functional connectivity of neurocognitive networks in children. Human Brain Mapping, 38(1), 97–108. 10.1002/hbm.23346 27534733 PMC5796541

[hbm24855-bib-0026] Patanaik, A. , Tandi, J. , Ong, J. L. , Wang, C. , Zhou, J. , & Chee, M. W. L. (2018). Dynamic functional connectivity and its behavioral correlates beyond vigilance. NeuroImage, 177, 1–10. 10.1016/j.neuroimage.2018.04.049 29704612

[hbm24855-bib-0027] Picchioni, D. , Pixa, M. L. , Fukunaga, M. , Carr, W. S. , Horovitz, S. G. , Braun, A. R. , & Duyn, J. H. (2014). Decreased connectivity between the thalamus and the neocortex during human nonrapid eye movement sleep. Sleep, 37(2), 387–397. 10.5665/sleep.3422 24497667 PMC3900615

[hbm24855-bib-0028] Pilcher, J. J. , & Huffcutt, A. I. (1996). Effects of sleep deprivation on performance: A meta‐analysis. Sleep, 19(4), 318–326. 10.1093/sleep/19.4.318 8776790

[hbm24855-bib-0029] Power, J. D. , Barnes, K. A. , Snyder, A. Z. , Schlaggar, B. L. , & Petersen, S. E. (2012). Spurious but systematic correlations in functional connectivity MRI networks arise from subject motion. NeuroImage, 59(3), 2142–2154. 10.1016/j.neuroimage.2011.10.018 22019881 PMC3254728

[hbm24855-bib-0030] Preti, M. G. , Bolton, T. A. W. , & Van De Ville, D. (2017). The dynamic functional connectome: State‐of‐the‐art and perspectives. NeuroImage, 160, 41–54. 10.1016/j.neuroimage.2016.12.061 28034766

[hbm24855-bib-0031] Rashid, B. , Blanken, L. M. E. , Muetzel, R. L. , Miller, R. , Damaraju, E. , Arbabshirani, M. R. , … Calhoun, V. (2018). Connectivity dynamics in typical development and its relationship to autistic traits and autism spectrum disorder. Human Brain Mapping, 39(8), 3127–3142. 10.1002/hbm.24064 29602272 PMC6045960

[hbm24855-bib-0032] Roweis, S. (1998). EM algorithms for PCA and SPCA. Advances in Neural Information Processing Systems, 10(10), 626–632.

[hbm24855-bib-0033] Samann, P. G. , Tully, C. , Spoormaker, V. I. , Wetter, T. C. , Holsboer, F. , Wehrle, R. , & Czisch, M. (2010). Increased sleep pressure reduces resting state functional connectivity. Magnetic Resonance Materials in Physics Biology and Medicine, 23(5–6), 375–389. 10.1007/s10334-010-0213-z 20473549

[hbm24855-bib-0034] Shao, Y. , Wang, L. , Ye, E. , Jin, X. , Ni, W. , Yang, Y. , … Yang, Z. (2013). Decreased thalamocortical functional connectivity after 36 hours of total sleep deprivation: Evidence from resting state FMRI. PLoS One, 8(10), e78830 10.1371/journal.pone.0078830 24205327 PMC3808277

[hbm24855-bib-0035] Smith, S. M. , Miller, K. L. , Salimi‐Khorshidi, G. , Webster, M. , Beckmann, C. F. , Nichols, T. E. , … Woolrich, M. W. (2011). Network modelling methods for FMRI. NeuroImage, 54(2), 875–891. 10.1016/j.neuroimage.2010.08.063 20817103

[hbm24855-bib-0036] Tagliazucchi, E. , & Laufs, H. (2014). Decoding wakefulness levels from typical fMRI resting‐state data reveals reliable drifts between wakefulness and sleep. Neuron, 82(3), 695–708. 10.1016/j.neuron.2014.03.020 24811386

[hbm24855-bib-0037] Tashjian, S. M. , Goldenberg, D. , Monti, M. M. , & Galvan, A. (2018). Sleep quality and adolescent default mode network connectivity. Social Cognitive and Affective Neuroscience, 13(3), 290–299. 10.1093/scan/nsy009 29432569 PMC5836271

[hbm24855-bib-0038] Wang, C. , Ong, J. L. , Patanaik, A. , Zhou, J. , & Chee, M. W. (2016). Spontaneous eyelid closures link vigilance fluctuation with fMRI dynamic connectivity states. Proceedings of the National Academy of Sciences of the United States of America, 113(34), 9653–9658. 10.1073/pnas.1523980113 27512040 PMC5003283

[hbm24855-bib-0039] Wong, C. W. , Olafsson, V. , Tal, O. , & Liu, T. T. (2013). The amplitude of the resting‐state fMRI global signal is related to EEG vigilance measures. NeuroImage, 83, 983–990. 10.1016/j.neuroimage.2013.07.057 23899724 PMC3815994

[hbm24855-bib-0040] Xu, H. , Shen, H. , Wang, L. , Zhong, Q. , Lei, Y. , Yang, L. , … Yang, Z. (2018). Impact of 36h of total sleep deprivation on resting‐state dynamic functional connectivity. Brain Research, 1688, 22–32. 10.1016/j.brainres.2017.11.011 29174693

[hbm24855-bib-0041] Yan, C. G. , Wang, X. D. , Zuo, X. N. , & Zang, Y. F. (2016). DPABI: Data Processing & Analysis for (resting‐state) brain imaging. Neuroinformatics, 14(3), 339–351. 10.1007/s12021-016-9299-4 27075850

[hbm24855-bib-0042] Yao, Z. J. , Liao, M. , Hu, T. , Zhang, Z. , Zhao, Y. , Zheng, F. , … Li, L. J. (2017). An effective method to identify adolescent generalized anxiety disorder by temporal features of dynamic functional connectivity. Frontiers in Human Neuroscience, 11, 492 10.3389/fnhum.2017.00492 29081741 PMC5645525

[hbm24855-bib-0043] Yeo, B. T. , Tandi, J. , & Chee, M. W. (2015). Functional connectivity during rested wakefulness predicts vulnerability to sleep deprivation. NeuroImage, 111, 147–158. 10.1016/j.neuroimage.2015.02.018 25700949

[hbm24855-bib-0044] Yoo, S. S. , Gujar, N. , Hu, P. , Jolesz, F. A. , & Walker, M. P. (2007). The human emotional brain without sleep ‐ a prefrontal amygdala disconnect. Current Biology, 17(20), R877–R878. 10.1016/j.cub.2007.08.007 17956744

[hbm24855-bib-0045] Zhang, C. , Baum, S. A. , Adduru, V. R. , Biswal, B. B. , & Michael, A. M. (2018). Test‐retest reliability of dynamic functional connectivity in resting state fMRI. NeuroImage, 183, 907–918. 10.1016/j.neuroimage.2018.08.021 30120987

