# Supplementary material for: Impact of acute sleep deprivation on dynamic functional connectivity states
Source: Hum Brain Mapp. 2019 Nov 4;41(4):994–1005. doi: 10.1002/hbm.24855 (PMC7268022; doi:10.1002/hbm.24855)
Supplement: Supplementary file 1 — Table S1 Statistical comparisons of neuropsychological variables among two sessions of rested wakefulness and 28 hr of sleep deprivation. [file HBM-41-994-s001.docx]

Supplementary materials

**Method and materials:**

Neuropsychological tests

During these two studies, we used a psychomotor vigilance task (PVT) to measure the subjects’ sustained attention ability before each MRI scanning (Dinges & Powell, 1985). This task asked the subject to response the lighting up of the battery lamp on a portable, handheld computer. Lapses in attention were defined as reactions longer than a limited reaction time (500 ms). Both the reaction speed and number of lapses are recorded and used to reflect their vigilance levels after sleep deprivation. Meanwhile, a conventional 3-back working memory task is employed to examine their working memory ability in our experiments. 3-back hit represents the response the stimulus that were presented the three earlier. Meanwhile, Karolinska Sleepiness Scale (KSS) is a 10-points self-report questionnaire that can measure the subjunctive’s sleepiness level at a particular time (Akerstedt & Gillberg, 1990). KSS is sensitive to the fluctuations of situational sleepiness and reflects the psycho-physical state level in the last 10 min.

Table S1 Statistical comparisons of neuropsychological variables among two sessions of rested wakefulness and 28 hours of sleep deprivation.

|  | Karolinska Sleepiness Scale | PVT | | Spatial 3-back hits | Letter 3-back hits |
| --- | --- | --- | --- | --- | --- |
|  |  | Speed (1/s) | Lapses |  |  |
| SD | 6.85±1.99 | 3.61±0.39 | 7.11±6.08 | 10.64±5.62 | 14.35±5.49 |
| RW1 | 3.46±1.56 | 3.99±0.36 | 1.15±1.52 | 15.85±4.28 | 17.08±3.10 |
| RW2 | 3.15±1.14 | 3.93±0.40 | 1.27±2.09 | 14.62±5.56 | 15.81±4.82 |
| SD vs. RW1 | < 0.001 | < 0.001 | 0.001 | < 0.001 | 0.02 |
| SD vs. RW2 | < 0.001 | 0.001 | 0.001 | 0.009 | 0.24 |
| RW1 vs. RW2 | 0.37 | 0.11 | 0.61 | 0.03 | 0.24 |
| F (2,11) | 32.04 | 22.42 | 18.28 | 14.62 | 3.17 |
| *p* value | < 0.001* | < 0.001* | < 0.001* | 0.001* | .06 |
| *η^2^* | 0.73 | 0.65 | 0.60 | 0.55 | 0.21 |

Abbreviation: PVT, psychomotor vigilance task; SD, sleep deprivation; RW, rested wakefulness.

* Statistically different at *p* < 0.05 level

Table S2 Statistical comparisons of neuropsychological variables between 52 hours of sleep deprivation and 14 hours of recovery sleep.

|  | Karolinska Sleepiness Scale | PVT test | | Spatial 3-back hits |
| --- | --- | --- | --- | --- |
|  |  | Speed (1/s) | Lapses |  |
| SD | 8.29±0.99 | 3.92±0.60 | 3.79±5.03 | 10.36±4.94 |
| RS | 2.14±0.95 | 4.75±0.67 | 0.38±0.75 | 13.14±4.88 |
| *t* | 14.27 | -5.68 | 2.5 | 4.89 |
| *p*-value | < 0.001* | < 0.001* | 0.026* | 0.15 |
| Cohen’s *d* | 4.96 | 1.52 | 0.78 | 0.56 |

Abbreviation: SD, sleep deprivation; RS, recovery sleep.

* Statistically different at *p* < 0.05 level


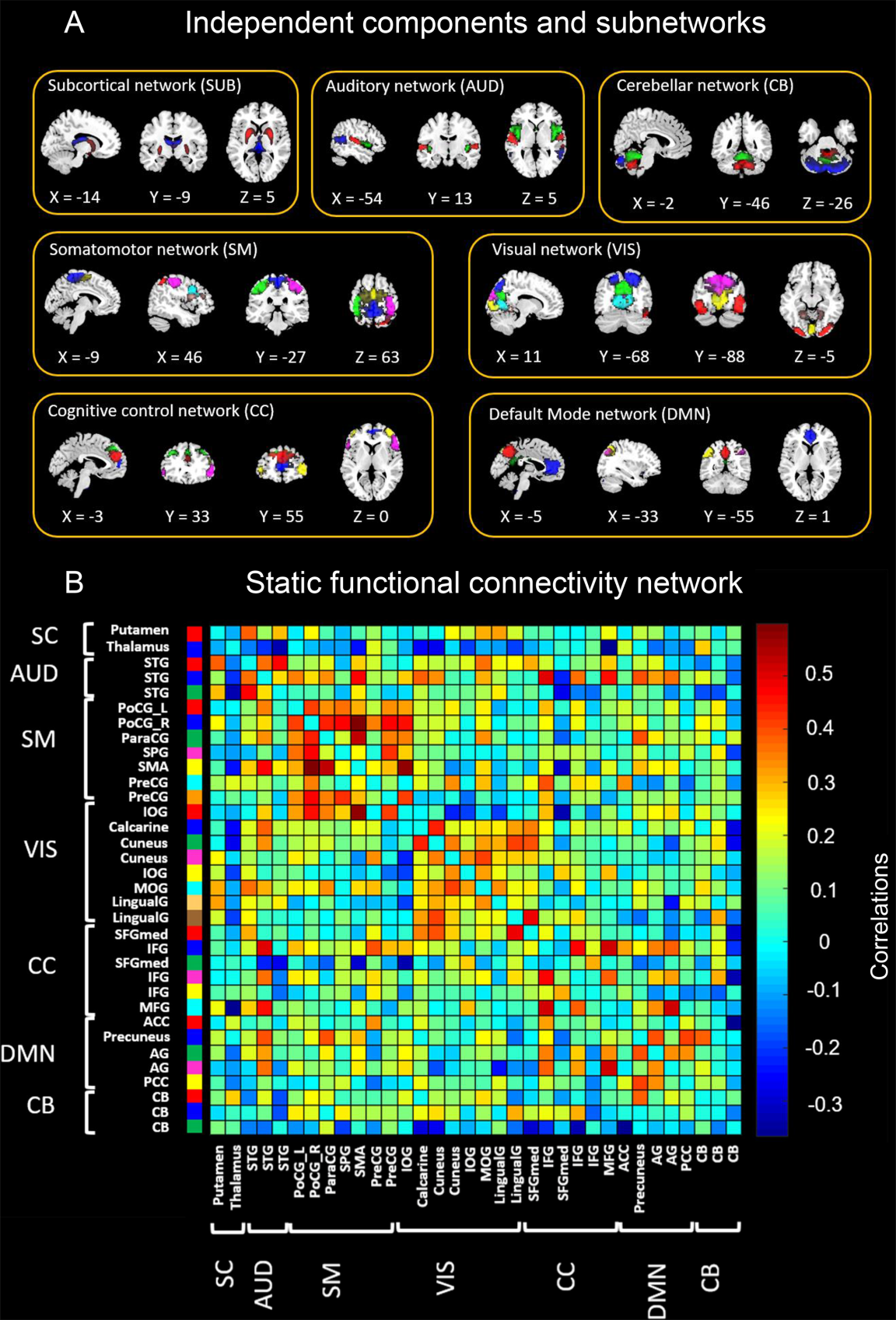


Fig. S1 Independent functional components and corresponding functional connectivity network for the 52 hours of sleep deprivation study.

Abbreviations: STG, superior temporal gyrus; PoCG, postcentral gyrus; ParaCL, paracentral lobule; SPG, superior parietal gyrus; SMA, supplementary motor area; PreCG, precentral gyrus; IOG, inferior occipital gyrus; MOG, middle occipital gyrus; MFGmed, middle frontal gyrus, medial part; IFG, inferior frontal gyrus; LingualG, lingual gyrus; MFG, middle frontal gyrus; ACC, anterior cingulate cortex; AG, angular gyrus; PCC, posterior cingulate cortex; CB, cerebellar.


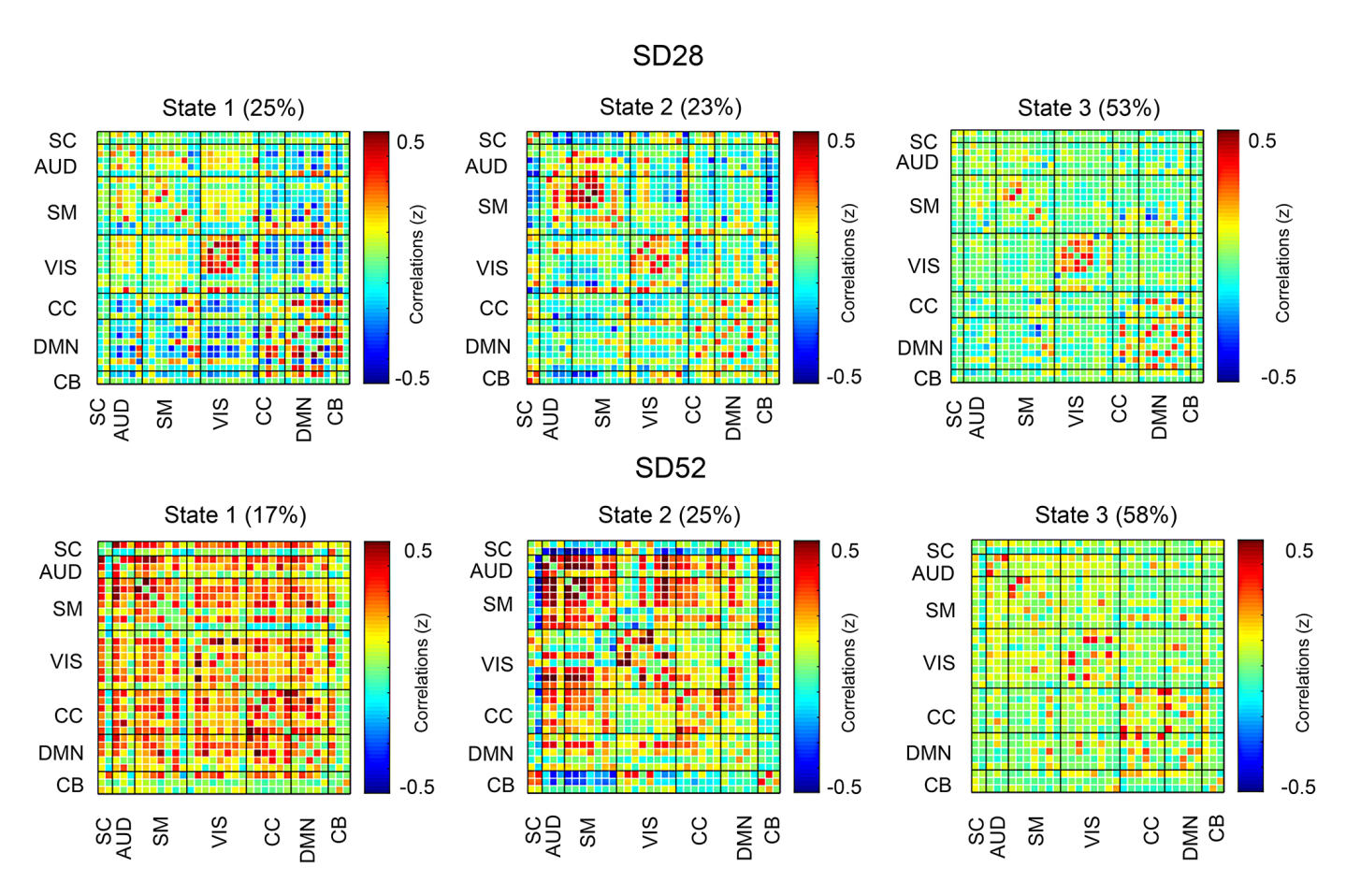


Fig. S2 Cluster centroids of brain states for 28 hours and 52 hours of sleep deprivation when sliding-window length = 30 TRs. The percentage means the occurrence probability of the specific brain state across the sliding windows of all subjects. Abbreviations: SC, subcortical network; AUD, auditory network; SM, somatomotor network; VIS, visual network; CC, cognitive control network; DMN, default mode network; CB, cerebella network.


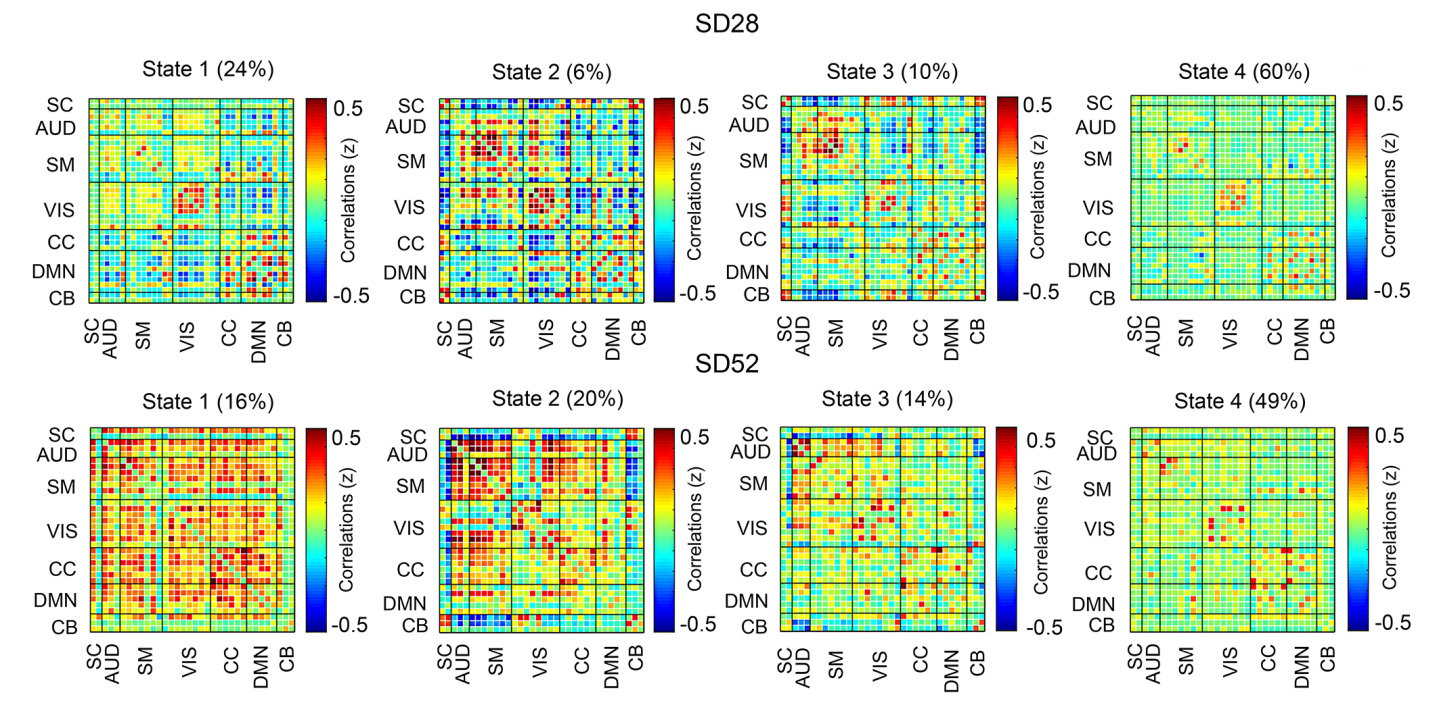


Fig. S3 Cluster centroids of brain states for 28 hours and 52 hours of sleep deprivation when number of clusters = 4. The percentage means the occurrence probability of the specific brain state across the sliding windows of all subjects. Abbreviations: SC, subcortical network; AUD, auditory network; SM, somatomotor network; VIS, visual network; CC, cognitive control network; DMN, default mode network; CB, cerebella network.


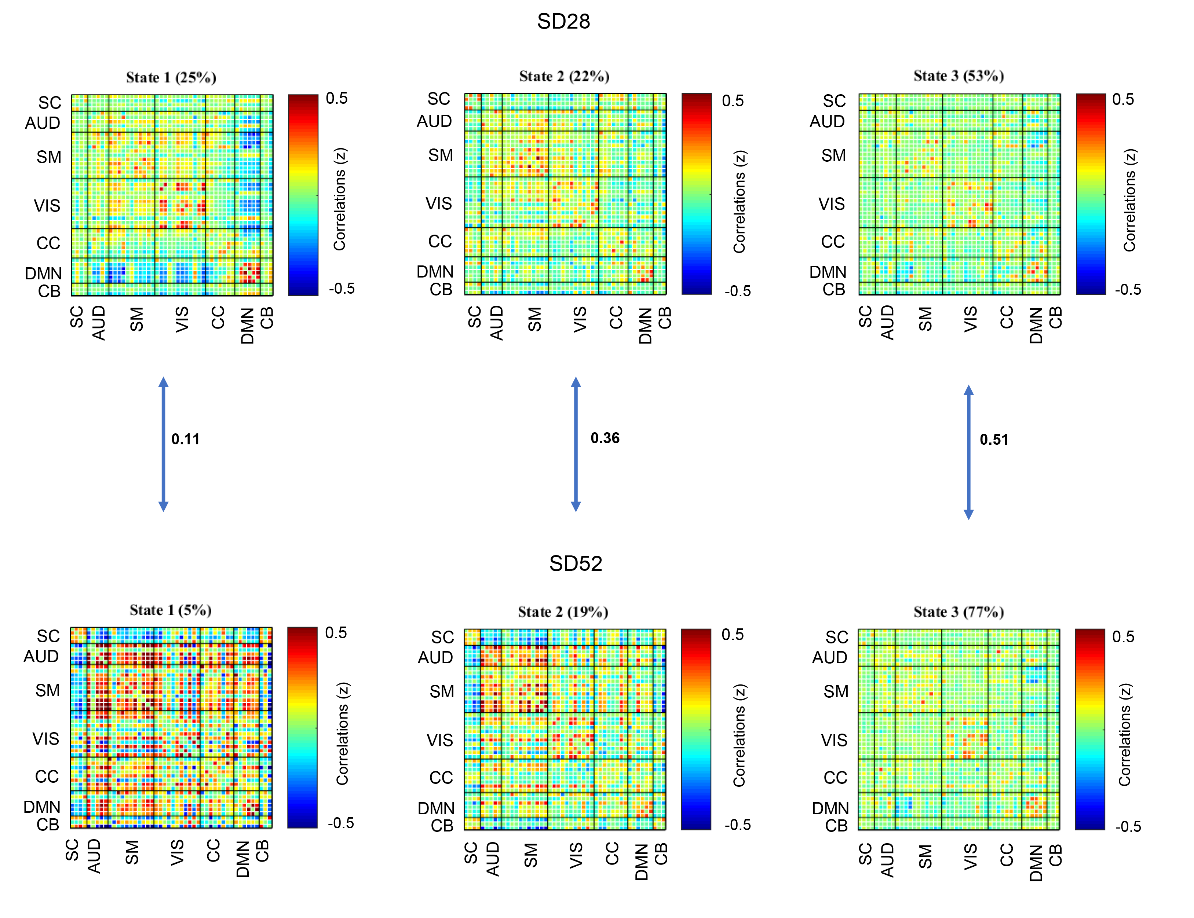


Fig. S4 Cross-correlations of dynamic connectivity states between 28 hours of sleep deprivation (SD28) and 52 hours of sleep deprivation (SD52) by using the same independent components and subnetworks.

Highest correlation is highlighted by bold line and bold value for each connectivity state.

Abbreviations: SC, subcortical network; AUD, auditory network; SM, somatomotor network; VIS, visual network; CC, cognitive control network; DMN, default mode network; CB, cerebella network.


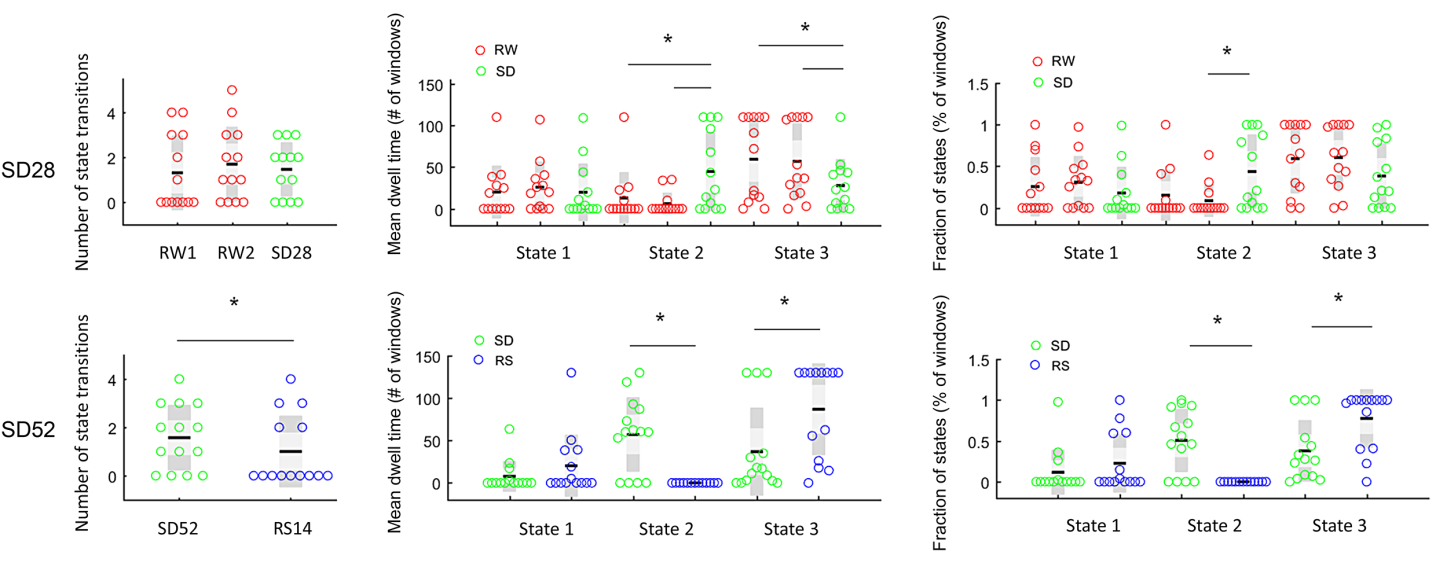


Fig. S5 Statistical comparisons of dynamic functional connectivity differences under different conditions of rested wakefulness (RW), sleep deprivation (SD) and recovery sleep (RS) when sliding-window length = 30 TRs.

Black line indicates the mean value of each group and light gray area represents the 95 % confidence interval.

* Statistical different at *p* < 0.05 level


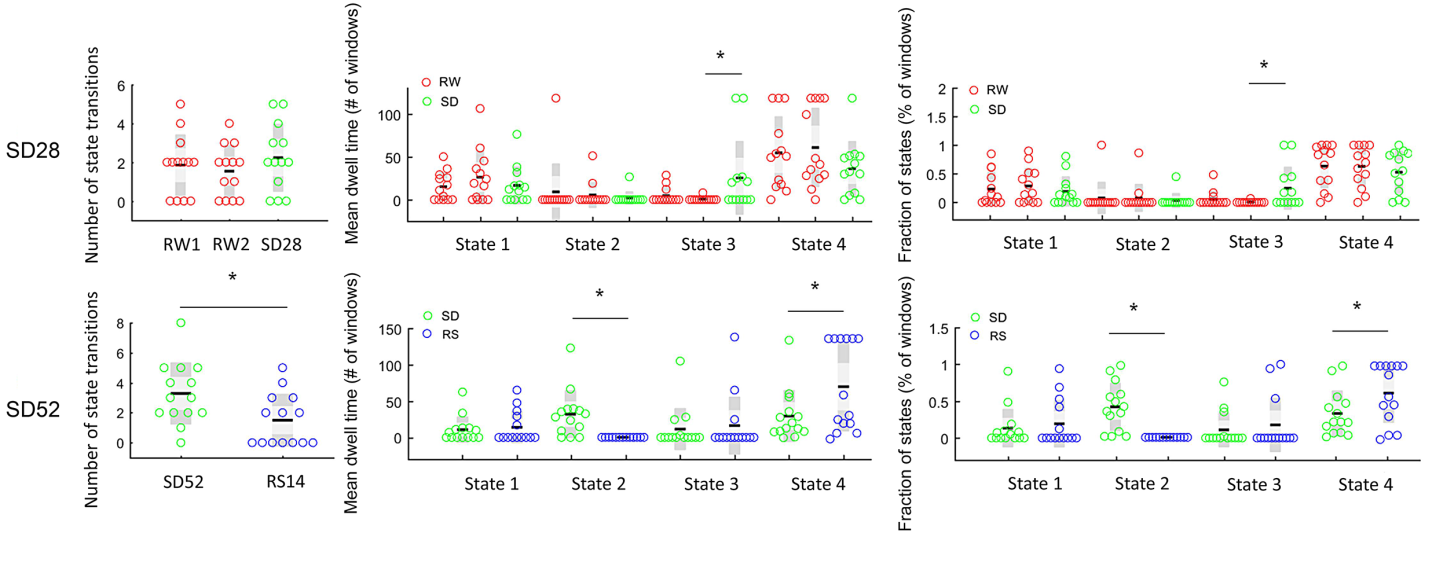
Fig. S6 Statistical comparisons of dynamic functional connectivity differences under different conditions of rested wakefulness (RW), sleep deprivation (SD) and recovery sleep (RS) when number of clusters = 4.

Black line indicates the mean value of each group and light gray area represents the 95 % confidence interval.

* Statistical different at *p* < 0.05 level


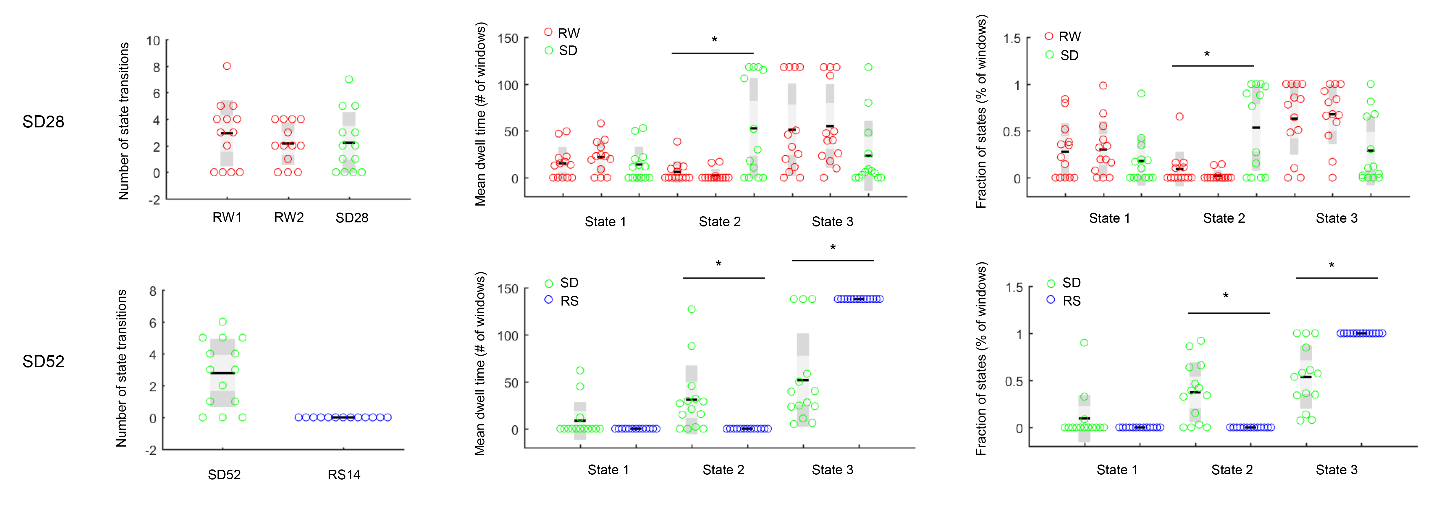


Fig. S7 Statistical comparisons of dynamic functional connectivity differences under different conditions of rested wakefulness (RW), sleep deprivation (SD) and recovery sleep (RS) by combing all resting state fMRI datasets.

Black line indicates the mean value of each group and light gray area represents the 95 % confidence interval.

* Statistical different at *p* < 0.05 level

Reference:

Akerstedt, T., & Gillberg, M. (1990). Subjective and objective sleepiness in the active individual. *Int J Neurosci, 52*(1-2), 29-37.

Dinges, D. F., & Powell, J. W. (1985). Microcomputer Analyses of Performance on a Portable, Simple Visual Rt Task during Sustained Operations. *Behavior Research Methods Instruments & Computers, 17*(6), 652-655. doi:Doi 10.3758/Bf03200977
